# Nanobiosensors for procalcitonin (PCT) analysis

**DOI:** 10.1002/jcla.25006

**Published:** 2024-01-24

**Authors:** Ahmad Mobed, Mohammad Darvishi, Amir Tahavvori, Iraj Alipourfard, Fereshteh Kohansal, Farhood Ghazi, Vahid Alivirdiloo

**Affiliations:** ^1^ Infectious and Tropical Diseases Research Center, Clinical Research Institute Tabriz University of Medical Sciences Tabriz Iran; ^2^ Infectious Diseases and Tropical Medicine Research Center (IDTMRC), Department of Aerospace and Subaquatic Medicine AJA University of Medical Sciences Tehran Iran; ^3^ Internal Department, Medical Faculty Urmia University of Medical Sciences Urmia Iran; ^4^ Institute of Biology, Biotechnology and Environmental Protection, Faculty of Natural Sciences Tehran University of Medical Sciences Tehran Iran; ^5^ Stem Cell Research Center Tabriz University of Medical Sciences Tabriz Iran; ^6^ Ramsar Campus Mazandaran University of Medical Sciences Ramsar Iran

**Keywords:** clinical application, nanomaterial, nanotechnology, procalcitonin

## Abstract

**Background:**

Procalcitonin (PCT) is a critical biomarker that is released in response to bacterial infections and can be used to differentiate the pathogenesis of the infectious process.

**Objective:**

In this article, we provide an overview of recent advances in PCT biosensors, highlighting different approaches for biosensor construction, different immobilization methods, advantages and roles of different matrices used, analytical performance, and PCT biosensor construction. Also, we will explain PCT biosensors sensible limits of detection (LOD), linearity, and other analytical characteristics. Future prospects for the development of better PCT biosensor systems are also discussed.

**Methods:**

Traditional methods such as capillary electrophoresis, high‐performance liquid chromatography, and mass spectrometry are effective in analyzing PCT in the medical field, but they are complicated, time‐consuming sample preparation, and require expensive equipment and skilled personnel.

**Results:**

In the past decades, PCT biosensors have emerged as simple, fast, and sensitive tools for PCT analysis in various fields, especially medical fields.

**Conclusion:**

These biosensors have the potential to accompany or replace traditional analytical methods by simplifying or reducing sample preparation and making field testing easier and faster, while significantly reducing the cost per analysis.

## Graphical abstract

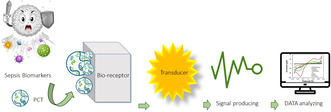

## 1 INTRODUCTION

Sepsis is a significant systemic inflammatory response due to infection caused by microbial pathogens such as bacteria, viruses, and fungi.^1^ If not carefully managed, sepsis can trigger a systemic inflammatory response, that, in severe cases, can lead to eventual death and organ dysfunction.^2^ Sepsis is a leading cause of death, and its frequency is growing worldwide, with a mortality rate of 40%–50% in low‐income countries and causing more deaths than breast, lung, and colorectal cancers combined.^3^ Therefore, there is an crucial need for a rapid, reliable, and simple method for diagnosing the cause of sepsis to improve the survival and treatment outcomes.^3^ The clinical manifestations of infections reflect the interactions between the host and microbes.^4^ An effective way to screen for infection is to monitor biomarkers involved in the host's immune response to reveal the severity of sepsis. On the other hand, reliance on a single biomarker to determine sepsis may lead to misidentification, as sepsis is the outcome of multiple infection problems.^5^ Du to sepsis, the body releases multiple triggers that disrupt normal blood flow, causing blood clots and leaking blood vessels.^6^ This deprives the organ of the nutrients it needs, leading to organ destruction. In severe cases, the heart weakens, blood pressure drops precipitously, and the patient goes into septic shock.^7^ Patients need accurate and rapid identification at this critical stage. A methodology for effective prognosis is to integrate a combination of early‐onset and late‐onset biomarkers to achieve a comprehensive patient sepsis profile. As already mentioned, sepsis is not only a multifactorial complication involving various factors, but also several biomarkers have been identified in association with sepsis. Each of these biomarkers has been evaluated in various studies in recent years.^8^, ^9^, ^10^ The Food and Drug Administration (FDA) approved the uses of C‐reactive protein (CRP) and procalcitonin (PCT) for the monitoring and diagnosis of sepsis in the clinical setting.^6^, ^7^ Even though PCT assays have presented encouraging results in the past decade, numerous limitations must be considered before using these tests in routine clinical practice. For example, serum PCT levels are increased in patients with non‐infectious diseases such as cancer, trauma, burns, and immunomodulatory treatments. An increase in PCT can also be expected in the presence of pro‐inflammatory cytokines, cardiogenic shock, and during the first 2 days of peritoneal dialysis treatment.^11^, ^12^ In addition, PCT levels were incorrectly increased in patients with varying degrees of chronic kidney disease, which could modify baseline outcomes and complicate identification of the underlying bacterial infection.^11^, ^12^ Therefore, physicians should therefore rule out the above scenarios to ensure that there are no confounding issues masking PCT measurements.^12^ Given the importance of multi‐marker detection, researchers recommend biosensors as a new method for determining the diagnostic value of PCT and early detection of sepsis. Biosensor technology enables clinicians to design effective patient treatment plans based on clinical profiles generated by disease prognosis.

## 2 PROCALCITONIN

**FIGURE 1:**
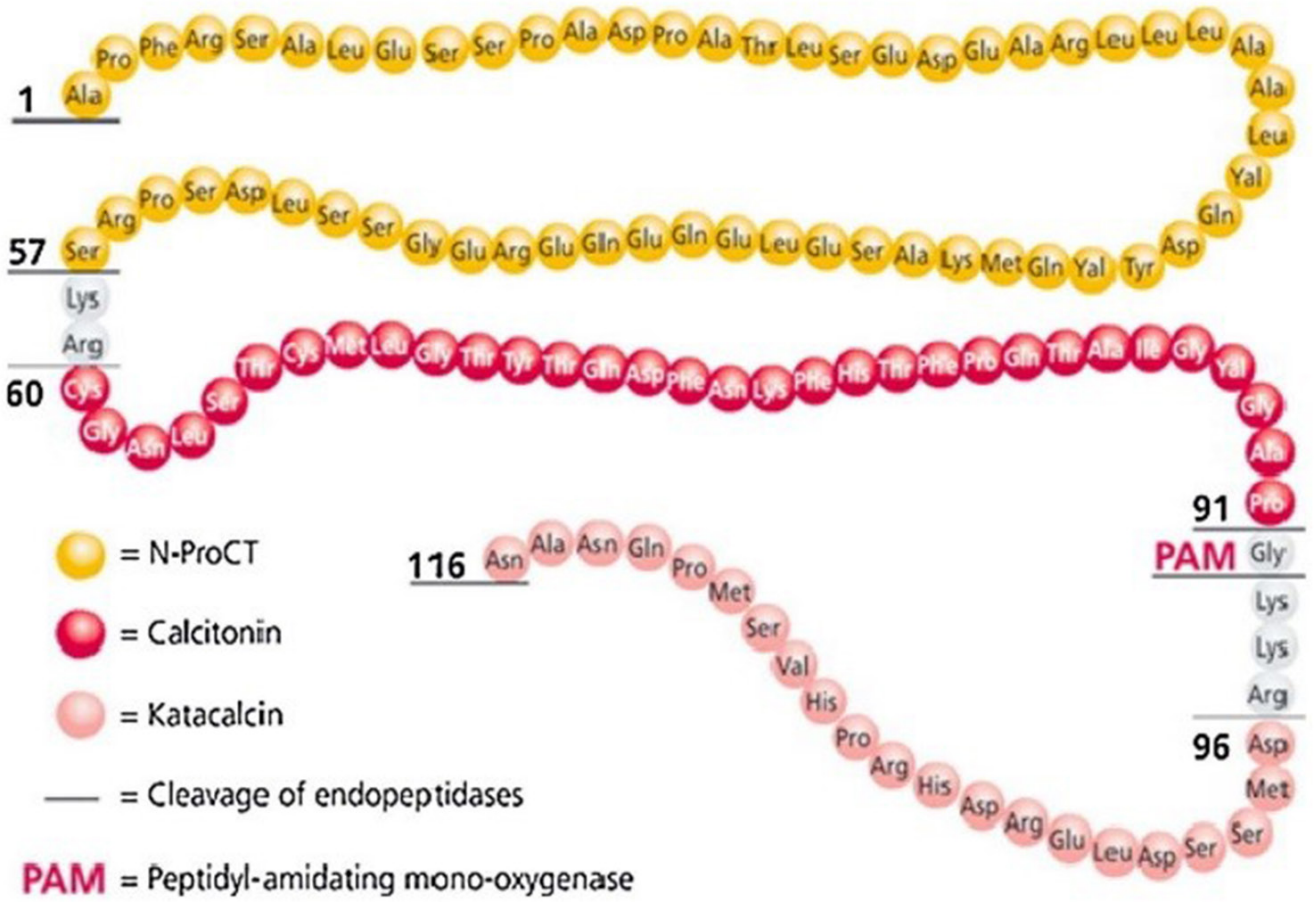
The structure and synthesis of PCT is the precursor of calcitonin and the site of occurrence of the calc‐1 gene on chromosome 11 of the human genome. After transcription of CT‐DNA into mRNA, the first translation product is pre‐PCT, which undergoes various modification steps to convert to PCT.^14^

PCT is a critical biomarker for bacterial infections and in particular for the monitoring of sepsis. Some exciting discoveries and latest developments in the scientific literatures propose that PCT is one of the paramount biomarker in the human body (Figure 1).^13^

In some cases, PCT levels may not increase as expected in bacterial infections. The aims for this observation may be diverse, and it must be stressed that the production of PCT can be prompted by host inflammatory cytokines and endotoxins.^13^ PCT is formed in response to not only to bacterial endotoxins but also to host inflammatory cytokines. Although the main mechanism is vague, it is recognized that lipoteichoic acid (LTA) of the Gram‐positive bacteria cell walls that are recognized by Toll‐like receptor 2 (TLR2) and toll‐like receptor 4 (TLR4) play a critical role.^15^ Patients infected with Gram‐positive bacteria have elevated levels of PCT, presumably as a result of an inflammatory cytokine response.^15^ Serum PCT levels have been revealed to increase 6–12 h after initial bacterial infection and increase steadily 2–4 h after the onset of sepsis.^16^, ^17^ The half‐life of PCT is 20–24 h. Consequently, an adequate host immune response and 24 h of antibiotic therapy result in a corresponding 50% reduction in PCT levels.^16^, ^17^

## 3 METHODS IN DETECTION OF PCT

In current clinical training, some analytical techniques have been industrialized to detect serum PCT levels with varying sensitivities (approximately 0.06 ng/mL). All presently existing evaluations for quantification of PCT are based on immunoassay methods. Amongst BRAHMS PCT LIA® (Thermo Fisher, Hennigsdorf, Germany) emerged as the first commercially current PCT assay that worked based on manual luminometric immunoassay.^18^ Additionally, BRAHMS PCT Kryptor appeared as a fast and more sensitive mechanized assay that is approved by the FDA in 2008 for use in identification of septic shock and severe sepsis.^18^ Traditionally, the enzyme‐linked immunosorbent assay (ELISA) frequently used to quantify antibodies, antigens, proteins, and glycoproteins in biological samples according to several published papers.^19^, ^20^ Considering the limitations and drawbacks of routine tests such as low sensitivity and specificity, high cost, and the need for professionals to interpret the results and carry them out, the development of modern and advanced methods has been one of the main goals of researchers in recent years. In the meantime, methods based on nanotechnology have received more attention. Biosensors are one of the prominent methods in this field, which have many advantages over routine methods. In the following section, biosensors will be examined.

## 4 BIOSENSOR METHODOLOGY

**FIGURE 2:**
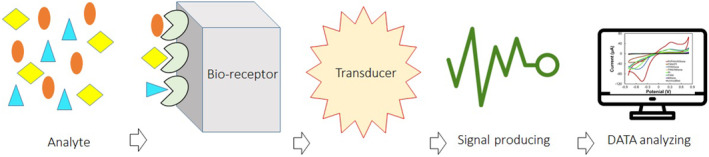
Simple structure of biosensor technology.^33^

Biosensor technology has made tremendous interdisciplinary contributions around the world. Biosensors have been extensively studied and developed in the medical, ecological, food, and pharmaceutical fields.^21^, ^22^ A biosensor is an integrated receptor–transducer device, which can convert a biological response into an electrical signal. Most transducers produce either optical or electrical signals that are usually proportional to the amount of analyte–bioreceptor interactions.^23^, ^24^ They are called ‘bio’ sensors because they make use of biological functions such as recognition and catalysis. Simple, fast, low‐cost, highly sensitive, and highly selective biosensors will contribute to next‐generation medical advances.^25^, ^26^ Biosensors are the most intensive research area in which disease recognition markers are emerging. Biosensors can be categorized into three groups according to the degree of integration of the separate components and the technique of attachment of the bio‐recognition or bio‐receptor molecule to the surface of the transducer.^27^, ^28^ In the first group, bioreceptors are physically encapsulated near the base sensor behind a discerning membrane, such as a dialysis membrane.^29^, ^30^ In the next group, immobilization will be performed via covalent binding at appropriately modified transducer interfaces or by incorporation into polymer matrices at transducing surfaces.^31^, ^32^ In the next group, immobilization will be via covalent binding at appropriately modified transducer interfaces or by incorporation into polymer matrices at transducing surfaces (Figure 2).^31^, ^32^

**FIGURE 3:**
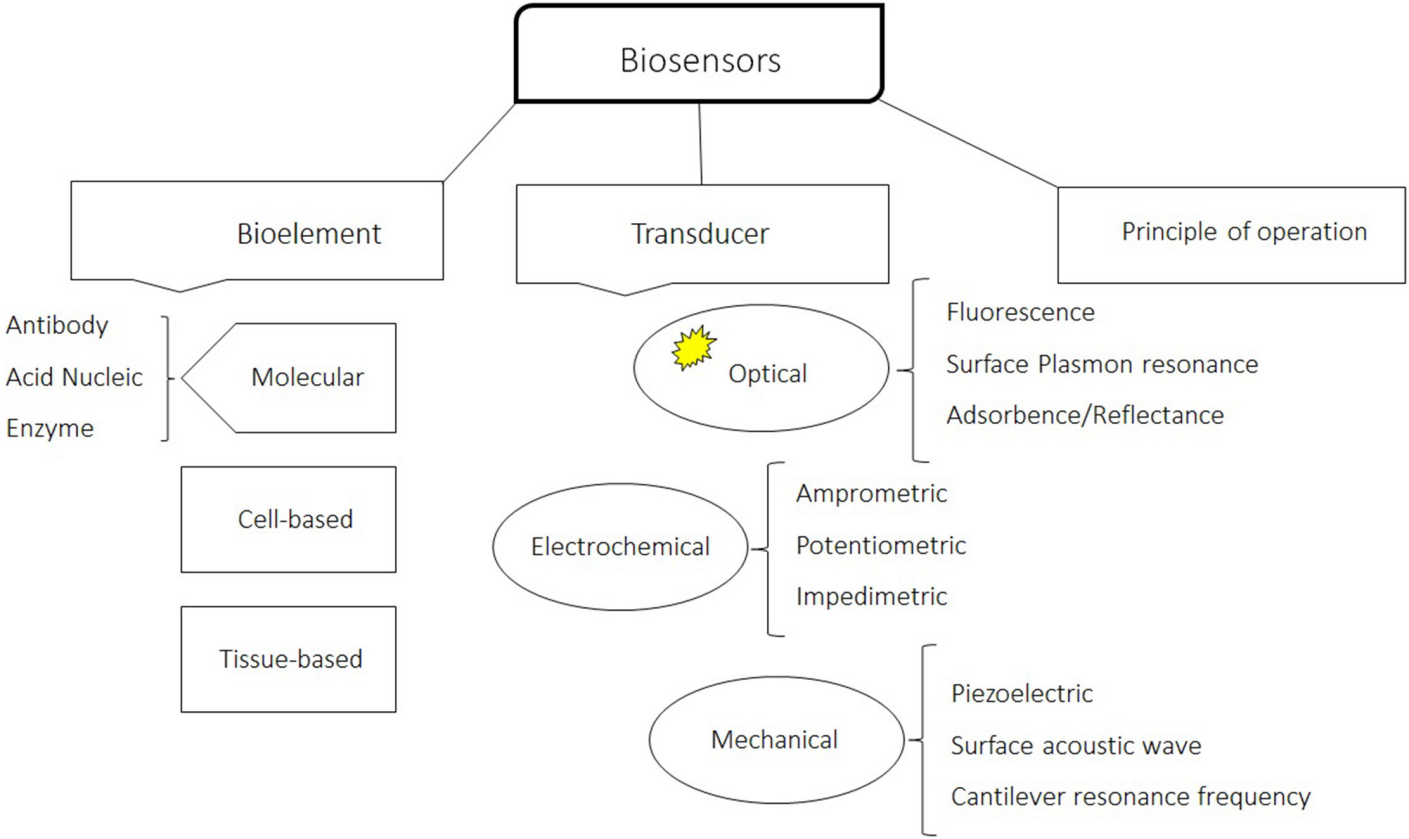
Biosensor classification.^39^

Additionally, biosensors can be categorized by the type of the physicochemical transducer or the type of the biorecognition element. Based on the transducer, biosensors can be divided into photoelectrochemical (optical), thermal, and piezoelectric biosensors.^34^, ^35^ Electrochemical biosensors include potentiometric biosensors (measuring the potential of a biosensor electrode relative to a reference electrode), conductometric biosensors (measuring changes in conductivity caused by a biochemical reaction), and amperometric biosensors (measuring the oxidation or reduction of an electroactive product or reactant).^36^, ^37^ Electrochemical biosensors are the most popular biosensors because they offer the advantages of good LOD, specificity, structural simplicity, and ease of operation. Recent advances in electronics enable these biosensors to be miniaturized as lab‐on‐a‐chip strategies for in vivo monitoring or as handheld devices for on‐site monitoring (Figure 3).^37^, ^38^

## 5 DEVELOPED BIOSENSORS FOR DETECTION OF PCT

**FIGURE 4:**
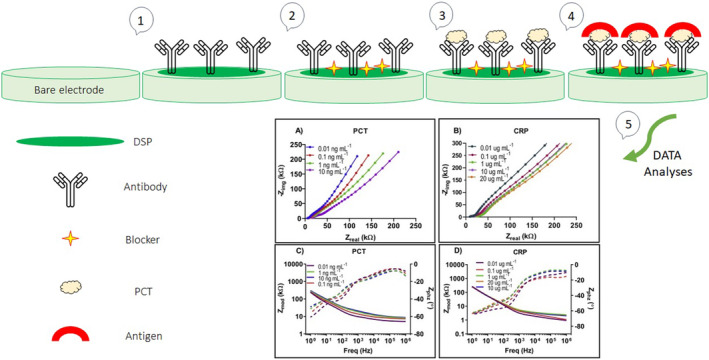
Schematic illustration of the immunoassay construction protocol for the PCT biosensing platform, adapted from ref.^40^

The latest achievement in the development of label‐free biosensors combines rapid detection of specific molecular markers, simplicity, ease of use, efficiency, accuracy, and cost‐effectiveness with the trend toward the development of wearable platforms being promising. A specific, highly sensitive, and low‐volume biosensor is further developed for rapid his PCT screening to detect sepsis. The developed biosensor is valuable as a sepsis screening tool for prognostic monitoring due to simultaneous dual‐marker detection by using the semiconductor active sensing electrode. Moreover, compared to previously described works, the developed system requires the smallest sample volume for optimal performance (Figure 4).^40^

**FIGURE 5:**
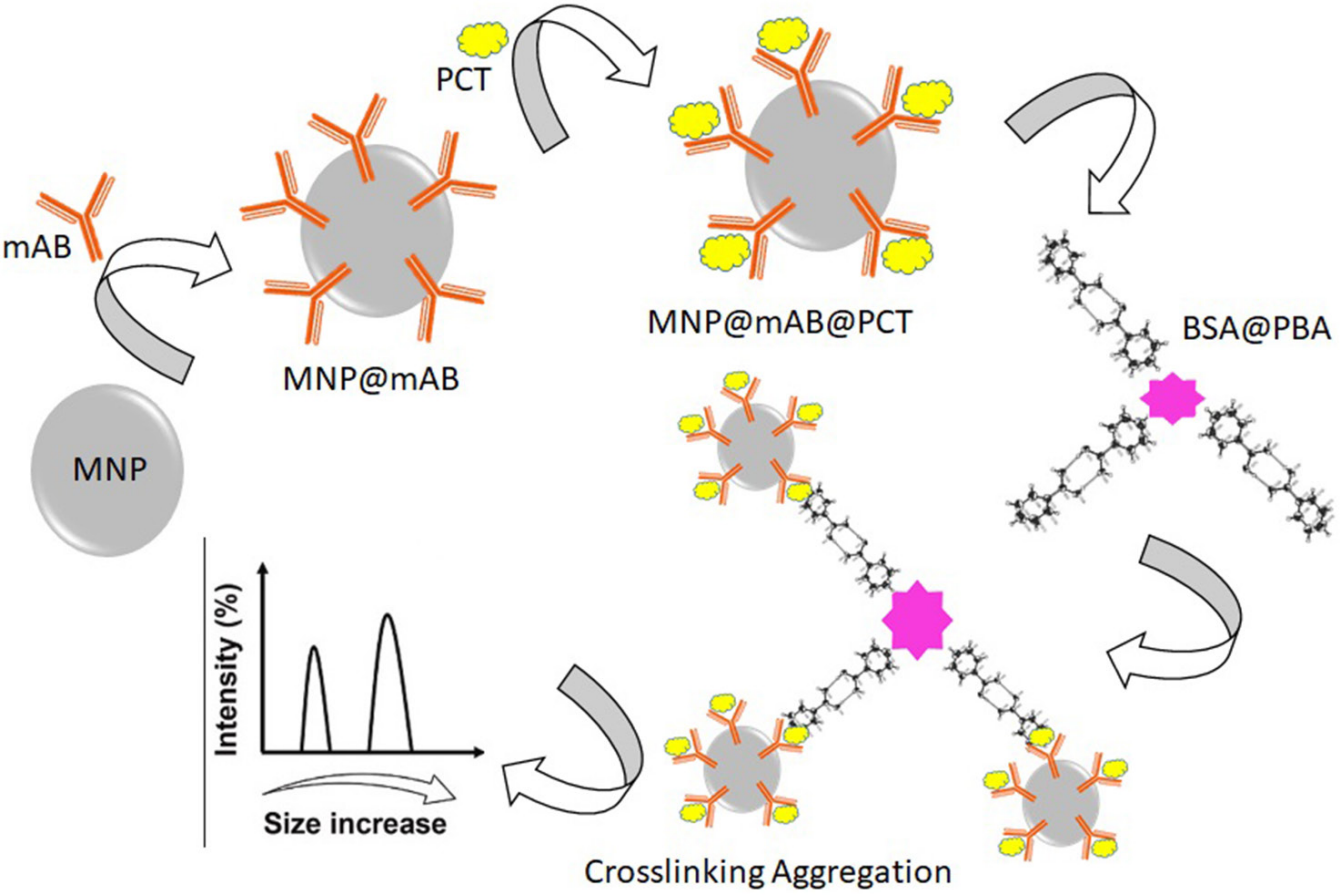
Developed immunosensor based on MNP for PCT determination, adapted from ref.^44^

An innovative label‐free electrochemical immunosensor was assembled for the quantitative detection of PCT using graphene‐coated CoNPs encapsulated in AuPtCu nano dendrites and 3D N‐doped carbon nano brushes (G‐Co@NCNBs). The developed immune device provides a valuable strategy for bioassay and immediate diagnosis of sepsis, and compared to similar systems, the nanocomposite developed in this study had a simple and low‐cost preparation method.^41^ PCT was determined with linearity and acceptable LOD using the electrochemical impedance spectroscopy (EIS) technique. The biosensor of the invention had good selectivity for PCT. The stability of the immunosensor was also examined for 1 month. The EIS developed in this study was an expensive technique to fabricate a point‐of‐care sensor for PCT compared to potentiostat methods such as differential pulse voltammetry. Therefore, trying to replace another technique in similar studies was suggested.^42^ A label‐free PEC immunosensor based on CdS sensitized BiVO_4_/GaON composite material was effectively advanced for determination of PCT. The developed biosystem displayed good stability and specificity, and serum sample analysis has been performed with acceptable results. Compared to most other detection methods for PCT, this immunosensor has a lower detection limit and a wider detection range.^43^ A unique enhanced DLS biosensor for boronic acid affinity detection enables ultra‐sensitive PCT detection. In this platform, monoclonal antibody‐modified magnetic nanoparticles (MNP@mAb) are planned as probes to capture his PCT from serum samples and generate DLS signals. As a result, this work expands the DLS biosensor to provide an advantageous and versatile approach for the rapid and sensitive detection of trace amounts of her PCT for a wider range of patients and more severe cases The main drawbacks of the developed sensors are the requirement of large sample volumes and long operating times, which pose problems for vulnerable patients such as infants and the elderly. The system developed in this study overcomes these limitations (Figure 5).^44^

**FIGURE 6:**
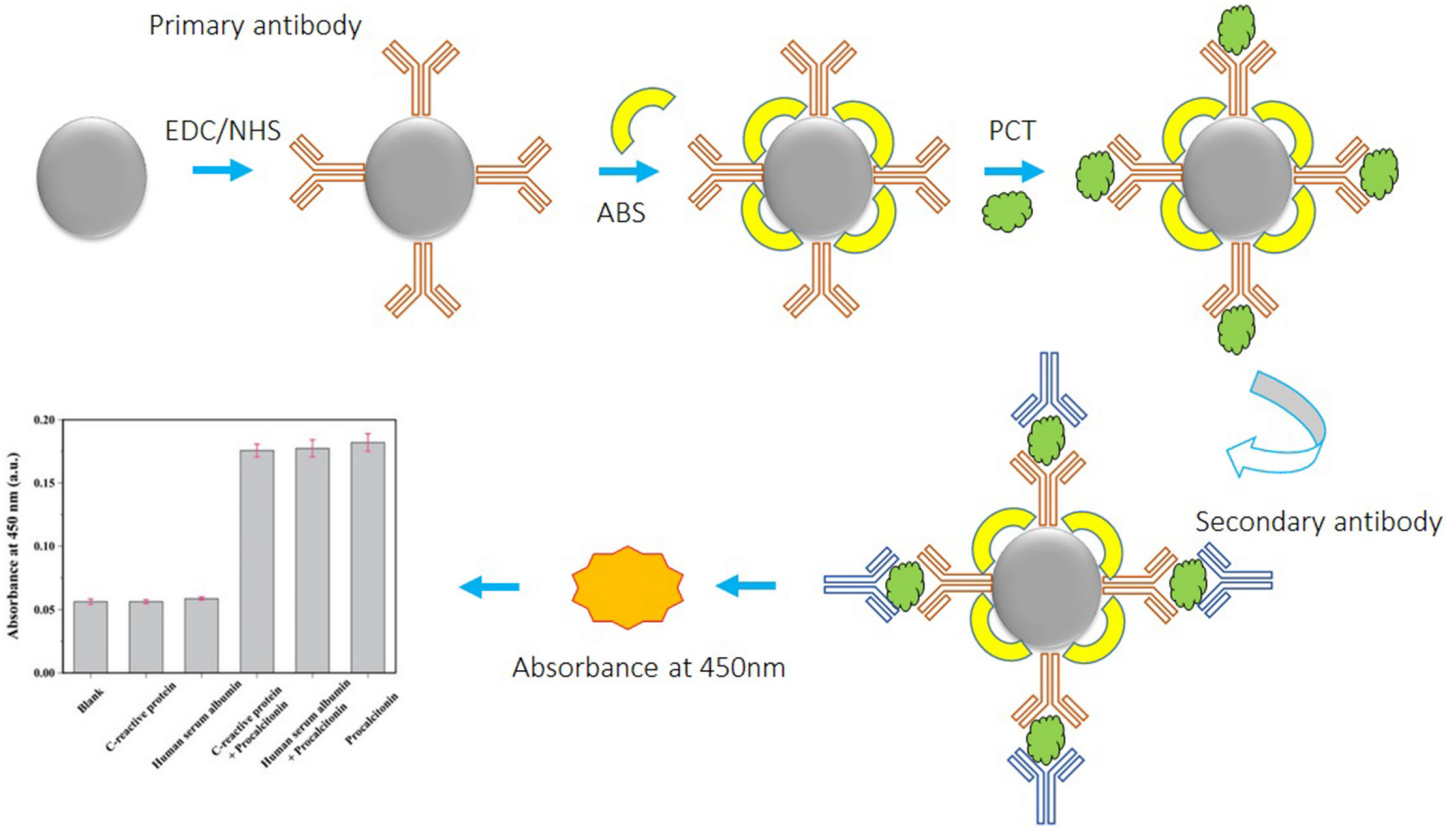
Representation of the colorimetric sensing platform for the detection of PCT, adapted from ref.^50^

The molecularly imprinted polymers (MIPs) have been proposed to detect PCT in animals. In this research, dopamine and norepinephrine as monomers were applied for production of MIP films on surface plasmon resonance (SPR) gold chips and evaluated the imprinting efficiency of animal PCT by analyzing its binding affinity, sensitivity, and selectivity to analytes. As a result of using the unique substrate and considering the versatility of these PNE‐based MIPs, they can be predicted as mimic receptors in widely used and disposable substrates such as ELISA micro‐well plates for the development of innovative antibody‐free assays.^45^ A new EC immunoassay was advanced for PCT antigen discovery based on the signal amplification plan of multiple nanocomposites. The created biosensor was constructed via layer‐by‐layer MWCNTs, coating graphene, chitosan, and glutaraldehyde composite on the working electrode. The electronic transfer rate and recovery of the surface area to capture a large number of primary antibodies (Ab1) were improved via the used material. In the other words, the highlight of this work is that graphene carbon nanotubes were successfully modified on the electrode, offering the electrical and chemical synergies for the development of a highly sensitive immunoassay format.^46^ A reduced graphene oxide (rGO)/gold nanoparticle (AuNP)‐based nanocomposite‐modified cellulose fiber paper‐based electrochemical biosensor was advanced for detection of PCT. The proposed biosensor also exhibited satisfactory selectivity and was able to detect PCT even in the presence of other intermediate electroactive species such as oxalate, glucose, and urea. Furthermore, the developed biosensor can be implemented as an improved and simple point‐of‐care (POC) assay approach for the initial analysis of PCT.^47^ A high‐sensitivity near‐infrared electrochemiluminescence biosensor based on Eu‐MOF with antenna effect and high efficacy catalysis of specific CoS_2_ hollow triple‐shelled nanoboxes was advanced for PCT determination. The fabricated sensor offers a practical technique for efficient and stable examination of systemic inflammatory response such as hepatitis B, terrible bacterial infection, and peritonitis. Due to the near‐infrared luminescence at 800–900 nm, the developed ECL sandwich biosensor was a safe system to avoid damage to real samples.^48^ Dual‐quenching ECL system g‐C_3_N_4_ to ZnONFs@PDA‐sCuO coupled with gold dendrite@polypyrrole core–shell nanoparticles based on resonance energy transfer (ECL‐RET) was advanced for PCT detection. The planned biosensor had suitable sensitivity, reproducibility, and specificity demonstrating the projected sensing technique could provide a good practical means and theoretical basis for the diagnosis of severe diseases. On the other hand, due to the used suitable nanocomposites, the perfect spectral overlap with the luminescent matrix achieves efficiency quenching through resonance energy transfer (RET).^49^ A colorimetric biosensor for the fast detection of PCT was established. The method involves the detection of PCT by immunomagnetic beads and a discovery antibody labeled with horseradish peroxidase to perform sandwich design, where it catalyzes the oxidation of 3,3′,5,5′‐tetramethylbenzidine to produce the colorimetric signal. In summary, the fabrication process of this sensor platform was simple and had great potential to detect sepsis and the severity of microbial infections through PCT detection in clinical samples (Figure 6).^50^

A high‐sensitive capillary immunosensor uniting biotin–streptavidin nano‐complex and porous‐layer surface modification signal amplification was advanced for sensing of PCT in serum. This work offers a robust and reliable method for developing capillary sensors that can be used in investigation of complex biologic samples. Compared with other capillary sensors, the sensitivity of the proposed PLOT‐SA sensor was significantly improved. This is due to the combination of two promising strategies: surface modification using a porous structured layer and a streptavidin–biotin–peroxidase nanocomplex signal amplification system.^51^ Plasmonic imaging of binding of single NPs to biomarkers captured on a sensor surface was settled as a fast digital immunoassay. The high contrast and fast plasmonic imaging lead to accurate counting of the individual biomarkers in acceptable linearity and LOD. In the developed system, the sensors can be prefabricated similar to the commercial SPR sensor chips, and digital counting of nanoparticles does not involve calibration of the optical setup.^52^ An innovative micromotor‐based fluorescence immunoassay (FMIm) was further developed for PCT identification. The micromotor combines self‐propulsion through magnetic induction (Ni layer) and catalytic generation of oxygen bubbles (PtNP inner layer) and the high binding capacity of specific antibodies on the polymer polypyrrole outer layer (PPy layer) to actively drive the PCT. In other words, the developed FMIm is highly sensitive and direct in clinical samples from very low birth weight infants (VLBWI) with suspected sepsis using small sample volumes (25 μL) at clinically relevant concentration ranges. This makes it possible to perform accurate PCT measurements.^53^ A fast and high‐sensitivity technique based on the fiber optic nanogold‐linked immunosorbent analysis was described for determination of PCT. This technique employs an immobilized capture probe on the fiber core surface of an optical fiber and a detection probe conjugated to AuNPs in a solution. Furthermore, the LOD of this method for PCT is lower than that of commercially available tests and is almost the lowest reported to date. Additionally, the 15 min analysis time meets his POCT requirements.^54^ The antibody‐based immunoplatform was engineered by AgNps and graphene for sensitive detection of PCT. The developed biosensor was biodegradable and can be employed in real samples.^55^ Most affinity peptides are cost‐effective and small in size for manipulation and mass production. A peptide‐based electrochemical biosensor was advanced for sensitive detection of PCT BP1. Even though this method requires additional evaluation for clinical use, the combination of electrochemical detection and high‐affinity peptide has numerous advantages over other bioanalytical methods. Environmental compatibility and low cost were the most prominent features of the designed sensor.^56^ A well‐ordered electrochemiluminescence sensing interface using peptide‐based specific antibody immobilizer and N‐(aminobutyl)‐N‐(ethylisoluminol) was fabricated for selective detection of PCT. The developed sensor showed acceptable sensitivity and wide‐range linearity. Although this approach requires further evaluation for clinical use, the combination of high‐affinity peptides and electrochemical detection has several advantages compared to other bioanalytical methods.^57^ An innovative peptide‐based biosensor was developed for ultra‐sensitive detection of PCT. In this sensor, the enzyme‐mimetic properties of iron nanocores within ferritin (Ft) generated abundant reactive oxygen species (ROS) in the presence of hydrogen peroxide, further enhancing the ECL signal. The planned system was effectively applied to measure the concentration of the PCT in human serum samples, indicating the potential use in clinical application. This biosensor showed a detection limit as low as 0.82 ag/mL in PBS, a 3‐fold improvement compared to the GFET biosensor.^58^ A high‐efficiency biosensor based on the ternary ECL system was created for PCT detection. Definitely, Ag NCs with stable luminescence properties were arranged with small‐molecule lipoic acid (LA) as the ligand, and its ECL radiation in persulfate (S_2_O8_2_–) was first described. Based on this, the developed biosensor showed high sensitivity for PCT detection, with a wide linear range (10 fg/mL–100 ng/mL) and a good LOD, which could be extended to clinical detection of multiple biomarkers.^59^ More details of the discussed biosensors are summarized in Table 1.

**TABLE 1:** Developed biosensors for determination of PCT.

Type	Platform	NPs	Matrix	Linear range	Operating time	LOD	Ref
Immunosensor	Non‐faradaic	ZnO	Serum	0.01–10 ng/mL	<15 min	0.10 ng/mL	40
Immunosensor	EC	AuPtCu NDs	Serum	0.0001–100 ng/mL	<30 min	0.011 pg/mL	41
Immunosensor	EC/EIS	AuNPs	Plasma	2.5–800 pg/mL	1 h	0.36 pg/mL	42
PEC	_	BiVO_4_/GaON/CdS	Serum	0.1 pg/mL–50 ng/mL	NA	0.03 pg/mL	43
Immunosensor	_	MNP	Serum	About 1 μL	<15 min	0.03 pg/mL	44
Immunosensor	MIPs, SPR	‐	Animal	‐	NA	15 ng/mL	45
EC	‐	MCM‐41	Serum	0.01–350 ng/mL	NA	0.5 pg/mL	46
EC	_	rGO‐AuNP	Serum	10–15 × 103 pg/mL	30 min	10 pg/mL	47
ECL	_	CoS2 TSNBs	Serum	10 fg/mL–100 ng/mL	1 h	3.65 fg/mL	48
ECL	ECL‐RET	ZnONFs@PDA	Biological	0.00005–50 ng/mL	24 h	17.2 fg/mL	49
EC	Colourimetry	HRP Ab2 þ TMB	Serum	0.1–10 ng/mL	<90 min	0.04 ng/mL	50
ECL	Capillary immunosensor	Silica capillary tubes	Serum	0.1 pg/mL–100 ng/mL	3 h	0.01 pg/mL	51
EC	Time‐resolved digital immunoassay	Gold‐coated glass	Serum	4.2 pg/mL–12.5 ng/mL	∼25 min	2.5 pg/mL	52
EC	Fluorescence immunoassay	Ni layer, PtNPs	Serum	0.5–150 ng/mL	Up to 30 min	0.07 ng/mL	53
Optical	FOPPR	AuNPs	Serum	1 pg/mL−100 ng/mL	≤15 min	95 fg/mL	54
EC	Peptide‐based	AgNp/SLG@ITO	Real	_	NA	0.55 ng/mL	55
EC	Peptide‐based	_	Serum	0.39 ± 0.11 nM	NA	12.5 ng/mL	56
ECL	Peptide‐based	PANI NRs/rGO‐Au	Serum	100 fg/mL‐ 50 ng/mL	NA	54 fg/mL	57
EC	CTS‐GFET	_	Serum	1 ag/mL–10 pg/mL	30 min	0.82 ag/mL	58
EC	Peptide‐based	α‐Fe_2_O_3_–Pt	Serum	10 fg/mL–100 ng/mL	1 h	3.56 fg/mL	59

Abbreviation: NA, Not available.

As mentioned before, biosensors are new and developing tools for detecting a wide range of biomarkers. Biosensors have many features and advantages such as high sensitivity and specificity as well as being affordable. Therefore, biosensors with high sensitivity, wide linear range, and low price are ideal systems. As indicated in the table, all discussed biosensors have acceptable and almost equal analytical characteristics, although some of them have better sensitivity and specificity. Nanomaterials used in the development of biosensors are important in several ways; for example, although gold‐based nanomaterials have high electrical conductivity, they are not affordable in terms of cost, while carbon‐based nanomaterials are inexpensive despite having good electrical conductivity.^60^ Therefore, we can identify cost‐effective biosensors based on the materials used in their development.

## 6 CONCLUSION, CHALLENGES, AND FUTURE PERSPECTIVES

Biosensors and nanoscale methods can be viewed as new promising tools that may lead to life‐saving treatment decisions and comprehensive knowledge of disease biomarkers. Having experienced the extreme challenges associated with traditional testing and validation methods, we believe we are well‐prepared when it comes to diagnostics to prevent serious illness. Based on studies, some paramount recommendations for biosensor development, including the mass industrialized FDA improvements, thoroughly satisfy structures such as low sample volume tests and disposability, and rapid improvement of nanotechnology should be deeply considered by researchers and commercial investors. We believe that the sensing mechanism will emerge with a multidisciplinary approach to enable rapid and point‐of‐care testing (POCT) of a wide range of biomarkers. PCT as a mediator of inflammation has recently become a specific marker for identifying severe bacterial infections, making it an ideal diagnostic indicator for systemic inflammation or specificity in organisms for major types of early‐stage bacterial infection and has received a lot of attention. Therefore, a new method of PCT measurement based on nanotechnology was established. In summary, biosensors aim to understand accurate diagnostics by developing tools that further advance the sensitivity, simplicity, specificity, multiplexing capability, affordability, and sustainability of ecosystems. Bioelectronics focuses on developing advanced materials and biosignal processing techniques for smart things.

## CONFLICT OF INTEREST STATEMENT

The authors declare no conflicts of interest.
